# Influence of Role Expectancy on Patient-Reported Outcomes Among Patients With Migraine: A Randomized Clinical Trial

**DOI:** 10.1001/jamanetworkopen.2024.3223

**Published:** 2024-04-24

**Authors:** Arne May, Gabriela F. Carvalho, Annika Schwarz, Hauke Basedau

**Affiliations:** 1 Department of Systems Neuroscience, University Medical Center Hamburg-Eppendorf, Hamburg, Germany; 2 Now with Department of Physiotherapy, Faculty of Health, Safety and Society, Furtwangen University, Furtwangen, Germany; 3 Now with Faculty of Social Sciences, University of Applied Sciences Bremen, Bremen, Germany

## Abstract

**Importance:**

It is usually assumed that an individual’s classification as a patient or a healthy person is determined by the presence or absence of disease, but little is known about whether the mere awareness of being a patient or a healthy control can play an important role for reporting outcomes.

**Objective:**

To investigate whether assignment to the role of a patient or a healthy control has an effect on patient-reported outcomes.

**Design, Setting, and Participants:**

This single center, double-blind, 3-group randomized clinical trial included consecutive patients from a tertiary headache clinic based at a single center in Germany who were invited to participate between October 2019 and June 2023. Statistical analysis was performed from January to March 2024.

**Intervention:**

Patients with migraine were randomized into 2 groups. The first group was told that this study was centered on migraine symptoms, whereas the second group was told that healthy controls were being sought for a study about patients with vertigo. A third group of age- and sex-matched headache-free participants served as controls. All participants viewed 2 standardized roller coaster videos and provided ratings of their perceived levels of motion sickness and dizziness.

**Main Outcomes and Measures:**

The primary outcome was self-reported vestibular symptoms. Secondary outcomes included differences in motion sickness, headache burden, and migraine disability. Outcomes were assessed using standardized questionnaires.

**Results:**

The final sample included 366 participants: 122 patients with migraine assigned the role of patient (MP) (migraine as patient): mean [SD] age, 37.56 [12.93] years; 105 [86.1%] female), 122 patients with migraine assigned the role of healthy participant (MH) (migraine as healthy): mean [SD] age, 37.03 [13.10] years; 107 [87.7%] female), and 122 headache-free controls (HC): mean [SD] age, 37.55 [11.56] years; 100 [82.0%] female). The assigned role of the individuals with migraine (MP vs MH) had a significant effect on self-disclosure of (1) estimation that symptoms (dizziness) will occur under specific conditions (self-reported vestibular symptoms: 79 MP [64.8%]; 29 MH [23.8%]; 9 HC [7.4%]; *P* < .001), (2) the increase of such symptoms (dizziness) after viewing the roller coaster video, and (3) the reported frequency (median [IQR] self-reported monthly headache days for MP: 7 [4-15] days; for MH: 5 [2-10] days; *P* = .008) and severity (median [IQR] migraine disability assessment score for MP: 35 [20-64] points; for MH: 25 [11-47] points; *P* = .005) of migraine symptoms. Statistically significant changes were also found for self-reported headache frequency and disability caused by migraine.

**Conclusions and Relevance:**

This randomized clinical trial found an effect of expectations regarding the role of a patient with respect to clinical and study outcomes. These findings suggest that role expectations should be taken into account when, for example, invasive treatments are discussed.

**Trial Registration:**

ClinicalTrials.gov Identifier: NCT06322550

## Key Points

**Question:**

Does the assigned role an individual accepts (ie, that of a patient vs a healthy control) influence clinical patient-reported outcomes?

**Findings:**

In this randomized clinical trial of 244 individuals with migraine, those who were randomly assigned to the role as a patient presented with significantly different clinical outcomes compared with patients who were assigned the role of a healthy control.

**Meaning:**

These results suggest that clinicians may need to be vigilant regarding the effect of expectations and the role of a patient with respect to study outcomes.

## Teaser

This randomized clinical trial investigates whether assignment to the role of a patient or a healthy control has an effect on self-reported outcomes among patients with migraine.

## Introduction

Migraine is a highly prevalent and disabling neurological disease with a negative effect on patients, their families, and society as a whole.^1,2,3^ Following the International Classification of Headache Disorders, 3rd edition (ICHD-3), a migraine is defined as headache attacks lasting from 4 to 72 hours with associated symptoms such as nausea, vomiting, and sensitivity to light and sound.^4^ Migraine is also frequently associated with motion sickness, vestibular symptoms, and abnormal motion and visual processing.^5,6,7^ The underlying pathophysiology of migraine is relatively well established, although not yet fully understood ^7^ Despite this, not all patients can be treated, and consequently, patient-reported outcome measures^8^ are becoming increasingly important in daily clinical practice and also randomized clinical trials.^9^ Next to this development, knowledge of modulating influences, such as context,^10^ expectation^11,12,13^ including placebo^14,15^ and nocebo effects,^16,17^ and susceptibility,^18^ on clinical management recently came into focus.

Little is known about the role of self-perception or the social role a patient fulfills when consulting a physician. Patients experiencing a disease will be expected to perform worse on tasks assessing function or to report a minimal degree of symptom severity while fulfilling this role.^19^ The question arises at to whether patients and healthy persons are not only distinguished by the presence or absence of the disease, but also by psychological components that are determined by the social role (ie, the knowledge of being a patient or a healthy control). Pivotal research work has shown that the knowledge (ie, the assignment of the role of being a patient) affects subjective pain reporting and even arithmetic tasks in healthy participants with mild seasonal allergic rhinitis.^19^ However, these effects have never been investigated in patients. Moreover, as the assignment of a role has an effect on cognitive measures, the question arises as to whether this role will also have an effect on the report of symptoms.

Focusing on the role that an individual takes on when presenting as a patient, we invited patients with migraine to take part in our study asking about the previous history of migraine and used a battery of validated health questionnaires, including the migraine disability assessment score (MIDAS). Given the presence of static and dynamic balance alterations among patients with migraine,^6,20^ we did not focus on the disease-defining headache in these patients but used vestibular symptoms to investigate role assignment, or more precisely, role expectancy. We also invited all participants to take part in a virtual roller coaster ride because patients with migraine reported more and longer symptoms of dizziness and motion sickness in a previous study.^21^ To investigate the effect of response bias, we randomly informed half of the patients with migraine that the study aims at investigating vestibular disorders and that they were invited as controls. We hypothesized that patients with migraine assigned the role of patients (MP) would present with more symptoms vs patients with migraine who were taking part in the study as migraine healthy controls (MH) and contrasted both groups with sex-matched and age-matched headache-free controls (HC) using the same protocol.

## Methods

This double-blind, 3-group randomized clinical trial was approved by the ethics committee of the Medical Association of Hamburg. All participants signed an informed consent form before enrollment. This study was preregistered with OSF in 2019 (https://osf.io/65e8f/) and, at the request of the editors, was also registered retrospectively with ClinicalTrials.gov. We followed the Consolidated Standards of Reporting Trials (CONSORT) reporting guideline.

Consecutive patients from a tertiary headache clinic based at the University Medical Center Hamburg-Eppendorf, Germany, were invited to participate between October 2019 and June 2022. They were screened and diagnosed with migraine by a headache specialist according to the ICHD-3.^4^ All migraine types (with aura, without aura, episodic, chronic) were eligible but any other type of headache and/or the presence of medication overuse were exclusion criteria. Additionally, participants without headache were recruited from the university hospital and the local community. None of the participants had a history of other neurological disease, self-reported diagnosis of vestibular disease, or history of trauma or pathology of the cervical spine (eg, whiplash-associated disorder).

### Study Design

We used a double-blind, 3-group, randomized clinical trial design. Two groups (patients with migraine) were randomized to a group allocation, and the third group (headache-free controls) was not randomized but otherwise treated identically. After agreeing to participate, migraine patients were randomly given the briefing information and therefore their group allocation. Physicians allocated the groups by pulling from a randomized, equally sized stock of flyers. Participants received the cover stories via the allocated flyer with study information and also by the physician from the outpatient clinic. Fifty percent of the patients with migraine were assigned to the MP group and received the information that they will take part in a migraine study (compared with healthy controls) and that this study would investigate migraine-associated dizziness by watching a visual stimulus (roller coaster video). The other 50% of patients with migraine were assigned to the MH group and received the information that this study was in cooperation with the university’s vertigo department and that they would take part as controls because they were healthy apart from their migraine. The study would investigate possible dizziness by watching a visual stimulus (roller coaster video). The sex-matched and age-matched HC group took part in the same experiment (not randomized) and received no additional information except that we were investigating the perception of a visual stimulus (roller coaster video). The protocol for the intervention was standardized and identical for all participants (Supplement 1, eFigure 1 in Supplement 2).

All participants were then investigated in a different building by an unrelated investigator blinded to the group allocation. The investigator was also not involved in the analysis of data. The patients handed the flyer over to the investigator (as they were laminated and were reused) after the experiment, and thus unblinding the investigator only after the study. All participants filled out a set of questionnaires regarding demographical and headache features including age, migraine onset, intensity, frequency, family predisposition of migraine, current headache and migraine disability (MIDAS score^22^). Furthermore, participants completed the Patient Health Questionnaire–8 questionnaire^23^ in order to screen for the presence of depression. The presence of vestibular symptoms was also assessed: patients were encouraged to describe their symptoms according to the Barany Classification of Vestibular Disorders^24^ including its form (internal or external vertigo, dizziness or postural symptoms), its duration, frequency, and concomitant occurrence with migraine attacks. Motion sickness susceptibility was tested using the Motion Sickness Susceptibility Questionnaire Short-form (MSSQ)^25^ and the disability level related to vestibular symptoms were assessed using the Dizziness Handicap Inventory (DHI-G).^26^ After filling out the questionnaires, all participants watched 2 standardized roller coaster videos that were validated in our previous studies.^21^ Participants rated the perceived motion sickness and dizziness using a numerical rating scale. Furthermore, they characterized the symptoms severity after each video using the Simulator Sickness Questionnaire.^27^ All questions regarding headache were filled out after the experiments and symptom ratings. To also reduce experimenter bias, the investigators did not wear white coats.

### Outcome

The main outcome was self-reported vestibular symptoms. Secondary outcomes were: (1) differences in standardized motion sickness questionnaires, (2) reporting differences in the intervention (roller coaster)-associated questionnaires, and (3) differences in headache burden and migraine disability questionnaires.

### Statistical Analysis

The power calculation for the main outcome was based on previous group differences regarding self-reported vestibular symptoms.^21^ Prior to preregistration a sample size calculation using G*Power version 3.1.9.3 for Mac (Mathematisch-Naturwissenschaftliche Fakultät, University Düsseldorf, Germany)^28^ was conducted. With an anticipated effect size of 0.36, a .5 α value and a power of 80%, and a group n allocation ratio 1:1 in Wilcoxon-Mann-Whitney test calculation resulted in 120 participants per group (eg, 3 groups = 360 participants in total) were required.

Baseline characteristics of patients and controls are detailed using means and SDs for continuous variables, and median values with IQRs when suitable. For categorical variables, frequency and percentages are used. Due to the nonnormal distribution of the continuous variables, group comparisons used the Kruskal-Wallis test. For pairwise comparison in the post hoc analysis, the Dunn test was used. For comparison of the 2 patient cohorts, the Mann-Whitney test was used; and for categorial data comparisons, the Pearson χ^2^ test was used.

A 2-sided *P* < .05 was considered statistically significant with adjustment for multiple comparisons using Bonferroni correction. Statistical tables and analyses were produced using SPSS version 27 (IBM) and Matlab version R2022a (The MathWorks Inc) from January to March 2024.

## Results

**Figure 1.**
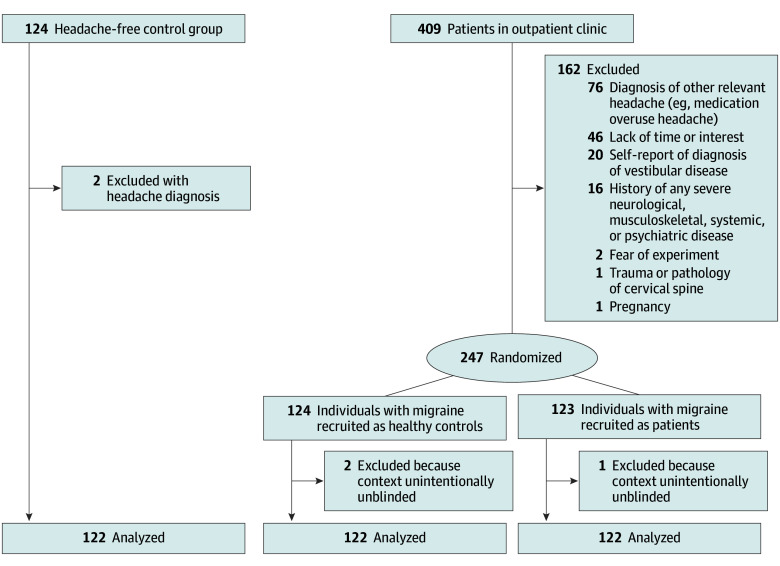
Study Flowchart Recruitment of patients with migraine took place through the outpatient clinic (n = 409 screened) where 162 patients were excluded due to various reasons; 247 patients were enrolled and randomly assigned to their role as a patient or a healthy control. In both groups, 3 patients were excluded. Headache-free participants (n = 124 screened) were recruited from hospital staff and the local community and 2 had to be excluded because of presence of a headache history. This resulted in 122 participants per group.

Between 2019 and 2023, 409 patients with migraine from the headache outpatient clinics were screened to participate in this study; 162 patients were excluded due to various exclusion criteria or lack of interest in participating (Figure 1). There were 247 patients who enrolled, 3 of whom had to be excluded for analysis as they were unintentionally unblinded. This left a total of 366 participants who were finally enrolled in this study: 244 patients with ICHD-3 diagnosis of migraine and 122 sex- and age-matched headache-free controls (mean [SD] age, 37.55 [11.56] years; 100 [82.0%] female). The 244 patients with migraine were randomized to MH and MP groups during enrollment, as aforementioned. Of these 244 patients enrolled, there were 122 patients with migraine in the MH group (mean [SD] age, 37.03 [13.10] years; 107 [87.7%] female) and 122 in the MP group (mean [SD] age, 37.56 [12.93] years; 105 [86.1%] female) (Figure 1, Table 1).

**Table 1.** Demographic Characteristics of All Participants

Participants characteristics	HC	MH	MP
No.	122	122	122
Sex, No. (%)			
Female	100 (82.0)	107 (87.7)	105 (86.1)
Male	22 (18.0	15 (12.3)	17 (13.9)
Age, mean (SD), y	37.55 (11.56)	37.03 (13.1)	37.56 (12.93)
Disease duration, mean (SD), y	NA	15.55 (11.84)	17.92 (12.42)
Migraine with and without aura, No.	NA	49	44
Migraine without aura, No.	NA	73	78
Chronic migraine (ICHD-3), No. (%)	NA	28 (23.0)	32 (26.2)
Episodic migraine (ICHD-3), No. (%)	NA	94 (77.0)	90 (73.8)

Abbreviations: HC, headache-free controls; ICHD-3, International Classification of Headache Disorders, 3rd edition; MH, participants with migraine who took part as healthy controls; MP, participants with migraine who took part as patients with migraine; NA, not available.

To verify that patients of the 2 migraine cohorts and headache-free controls did not differ, the following tests were performed: the χ^2^ test revealed no significant differences in sex and age between the 3 groups; the Mann-Whitney-Test between groups MH and MP showed no significant results for disease duration (*U* = 6215.00, *z* = −1.62; *P* = .10). The clinical diagnosis made by the assigning physician: chronic migraine with and without aura, chronic migraine without aura, episodic migraine with and without aura and episodic migraine without aura also showed no significant difference (χ^2^_3_ = 2.089; *P* = .55).

### Questionnaires

**Figure 2.**
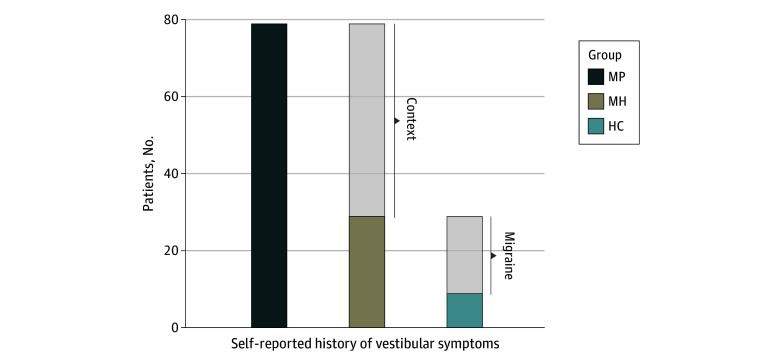
Self-Reported History of Vestibular Symptoms Figure shows the number of the total participants answering the questions to prior history of vestibular symptoms with “yes.” HC indicates headache-free controls; MP, patients with migraine recruited as patients; MH, patients with migraine recruited as healthy controls. The difference between the HC group and patients with migraine and the effect of role expectancy is shaded.

All study participants were asked to answer whether they commonly had vestibular symptoms before the actual intervention of the roller coaster videos. Figure 2 shows a significant association between group assignment and the answer (yes or no) to this question regarding vestibular symptoms (79 MP [64.8%] answered yes; 29 MH [23.8%] answered yes; 9 HC [7.4%] answered yes; χ^2^_4_ = 97.64, *CC* = 0.459, *P* < .001; Cramer* V* = 0.517; *P* < 001) (Table 2).

**Table 2.** Outcome of Vestibular Symptoms, Dizziness, and Depression Questionnaires for HC, MH, and MP Groups

Symptom questionnaires	HC	MH	MP	*P* value
Self-report of yes for vestibular symptoms, No. (%)	9 (7.4)	29 (23.8)	79 (64.8)	*P* < .001^a^
Motion Sickness Susceptibility Questionnaire, median (IQR), total score	6 (2-12)	10 (4-21)	13 (4-21)	*P* < .001^b^
Patient Health Questionnaire–8, median (IQR), total score	3 (2-5)	5 (3-8)	7 (3-9)	*P* < .001^b^
Dizziness Handicap Inventory, median (IQR), total score	0 (0-3)	0.5 (0-12)	10 (2-26)	*P* < .001^b^

Abbreviations: HC, headache-free controls; MH, patients with migraine who took part as healthy controls; MP, patients with migraine who took part as patients with migraine.

^a^ Tested by χ^2^ test.

^b^ Tested by Kruskal-Wallis test.

The median (IQR) MSSQ total score showed a significant difference across the 3 groups (MP: 13 [4-21] points; MH: 10 [4-21] points; HC: 6 [2-12] points; *H* = 16.15; *P* < .001) with the patients who took part as patients with migraine scoring highest in all comparisons (eTable 1 in Supplement 2). Post hoc Dunn test showed significant results comparing participants without headache and patients with migraine who took part as healthy controls (HC vs MH: *z* = 42.14; *P* = .004, Bonferroni corrected) and headache-free participants and patients with migraine who took part as patients (HC vs MP: *z* = 50; *P* = .001, Bonferroni corrected) but not for patients with migraine who took part as healthy controls and patients with migraine who took part as patients (MH vs MP: *z* = 50; *P* > .99, Bonferroni corrected). The median (IQR) DHI questionnaire score also showed a significant difference across all 3 groups (MP: 10 [2-26] points; MH: <1 [0-12] points; HC: 0 [0-3] points; *H* = 60.19; *P* < .001), and the post hoc comparison showed a significant difference comparing each group: HC < MH (*z* = 45.5; *P* = .001, Bonferroni corrected), HC < MP (*z* = 98.83; *P* < .001, Bonferroni corrected), and MH < MP (*z* = 53.32; *P* < .001, Bonferroni corrected).

### Rating of the Visual Intervention

**Figure 3.**
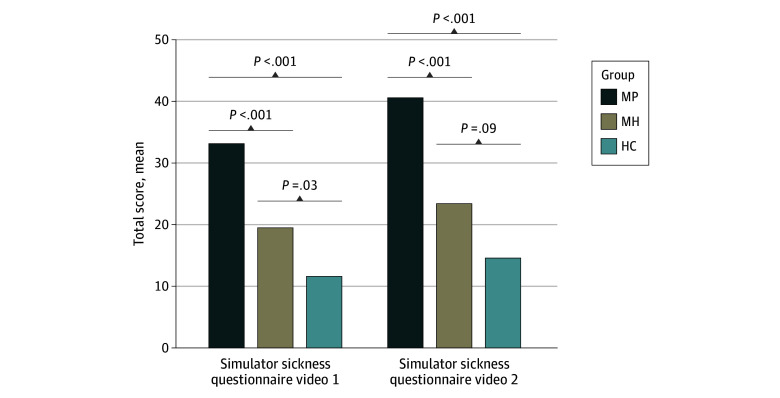
Simulator Sickness Questionnaire Results With Cohort Context After Vestibular Stimulation Figure shows the mean of the total score in the Simulator Sickness Questionnaire for the first roller coaster video (left) and the second rollercoaster video (right). HC indicates headache-free controls; MP, patients with migraine recruited as patients; MH, patients with migraine recruited as healthy controls.

Participants were presented 2 standardized roller coaster videos.^21^ Here, a significant group difference was found in the evaluation of the median (IQR) sum score of the Simulator Sickness Questionnaire video 1 (MP: 26.18 [11.22-48.62] points; MH: 14.96 [3.74-22.44] points; HC: 7.48 [3.74-14.96] points; *H* = 51.14; *P* < .001). Post hoc Dunn test showed significant results comparing HC vs MH (*z* = 38.59; *P* = .03, Bonferroni corrected) and HC vs MP (*z* = 95.14; *P* < .001, Bonferroni corrected) and again between patients with migraine (MH vs MP: *z* = 59.56; *P* < .001, Bonferroni corrected) (Figure 3).

The second roller coaster video^21^ (Simulator Sickness Questionnaire video 2 (median [IQR] MP, 29.92 [11.22-60.78]; median [IQR] MH, 14.96 [3.74-29.92]; median [IQR] HC, 7.48 [3.74-22.44] years; *H* = 48.92; *P* < .001) showed in the post hoc pairwise comparison that there was no significant difference between HC vs MH (*z* = 29.02; *P* = .09, Bonferroni corrected) whereas all other comparisons differed significantly from each other: HC vs MP (*z* = 92.03; *P* < .001, Bonferroni corrected) and MH vs MP (*z* = 63.02, *P* < .001, Bonferroni corrected) (Figure 3).

### Headache Questionnaires

The self-reported headache history questionnaire and Migraine Disability Assessment (MIDAS) were given to the patients after the visual stimulation, which unblinded them to the actual purpose of the study. Despite randomized grouping and no differences in the migraine groups based on physician medical diagnosis, we found significant differences in the median (IQR) self-reported monthly headache frequency (MP: 7 [4-15]; MH: 5 [2-10]; Mann-Whitney test: *U* = 5828.5, *z* = 2.84, *P* = .008). Likewise, the median (IQR) MIDAS score differed significantly between the groups MH vs MP (MP: 35 [20-64]; MH: 25 [11-47]; Mann-Whitney test: *U* = 5828.5, *z* = −2.84, *P* = .005) (eFigure 2 and eTable 2 in Supplement 2).

## Discussion

All clinical symptoms of migraine are subjective and solely rely on verbal report from the patient. Although there is no single objective test to diagnose migraine or to verify the severity of any of these symptoms, several validated questionnaires exist that help provide a more accurate diagnosis and improve the management of these conditions. The question arises whether these subjective self-assessments are subject to role expectation and thus may change depending on the social role the concerned person takes on.

Focusing on this issue, the current study found that the assigned role that the patient adopts has a significant effect on self-disclosure of (1) the reported frequency and severity of migraine symptoms, (2) estimation that symptoms (vestibular symptoms, dizziness) will occur under specific conditions, and (3) the increase of such symptoms (vestibular symptoms, dizziness) in a provocation study. These changes are rather robust and even affected MiDAS, which is a widely used and validated tool for assessing the disability caused by migraine and recommended by several headache societies.^22^ An additional finding was that a cornerstone of self-reported migraine severity and important outcome item for migraine studies (ie, the frequency of monthly attacks) differed significantly between patients with migraine who took part as patients with migraine from patients with migraine who took part as healthy controls. The striking feature here is that patients were asked for migraine severity and frequency only after unblinding. This means that patients of both groups were loyal to the adopted role as a patient or a healthy control despite debriefing. This reporting bias could well affect clinical outcome measures where, for example, the difference between medications and placebo effects is small.

Patients with migraine could behave how they did as a consequence to a variant of stereotype threat,^29^ because it is accepted that patients with migraine feel more dizziness, they report more dizziness in the past and also feel more dizziness in the roller coaster ride as part of a self-fulfilling prophecy. Interestingly, the threat here comes from attributing oneself to the stereotype. In other words, different than, for example gender, the situational predicament stems from artificial attribution to the stereotype “migraine patient” or “a human in good health.” It is important to note that patients did not overestimate or underestimate their headache frequency as such, but rather adopted the role assignment which consequently resulted in a matching self-assessment due to role expectancy.

These current findings presumably mirror a general human behavior that is independent of medicine and the definition of healthy or diseased. It certainly applies to all diseases where clinical assessment depends on subjective and poorly operationalized statements. Dizziness, pain, nausea, and sensory disturbances are highly subjective and may imply neurological disorders that are currently diagnosed based on clinical observation. Given that focusing on pain enhances pain perception,^30^ all migraine symptoms may be subject to focused attention and therefore susceptible for the effect described here. However, the core social process of clinical practice is the clinician-patient interaction. This particular social environment plays a significant role in shaping treatment expectations and even encompasses its own set of expectations within and beyond the clinical setting. It is important to underline that the context and resulting expectations affect the efficacy of treatments and subsequently affect an individual’s perception in a manner that they behave in a group-compliant manner.^31^

### Limitations

This study had limitations. MP patients received the information that the study investigates migraine-associated dizziness, so it is possible that the symptoms between the MP and MH groups might not only be due to the general effects of being a patient but to more specific effects induced by the information about migraine-associated dizziness. The latter specific nocebo effects might confound the general effects of being a patient, although this is difficult to disentangle. In this respect it is important to highlight that the groups did not differ regarding demographic variables, including depression, and the observed effects are most likely role-specific and not disease-specific and/or symptom-specific. Another limitation of this study is that we only assessed baseline measures of clinical characteristics in the patient groups, such as the headache type, attack frequency, and treatment characteristics. Ideally, all baseline measures (ie, including all questionnaires and even viewing the videos) would be assessed before randomization. This concerns particularly the patients with migraine who took part as healthy controls, because data from patients with migraine who took part as patients could be considered as baseline measures. As it is unpractical and indeed would involve a time effect, a session effect, and probably even learning effects, we decided against this process flow. Additionally, the randomization was not concealed (because it was a stack of flyers), which means it could theoretically have been subverted. However, the clinicians who handed the flyers to the patients were otherwise not involved in the study whatsoever, and there was no reason or incentive for them to put patients into a specific group.

## Conclusions

This study’s findings highlight the importance of being vigilant to the effect of suggestion on study outcomes and should be subject to further discussion in the scientific community as part of the discussion about suboptimal experimental methodology, publication bias, and the need for replication. The current findings do not preclude asking for attack frequency as a measure of migraine severity or using MIDAS to measure the disability caused by migraine. They merely raise the question whether and how much the context and/or social role bias the outcome. If the findings are also consistent in other diseases, they probably cannot be avoided in clinical practice but should be known and investigated and also taken into account when, for example, invasive treatments are discussed on the ground of such measures. Effective measures may include implementing active control groups that receive interventions and potentially blinding experimenters to the specific experimental condition being investigated, particularly when experimenters are responsible for evaluating participants’ performance or progress.

## Notes

Supplement 1.
Trial Protocol

Supplement 2. **eTable 1.** Ratings of the Visual Intervention (Roller Coaster Video) for Headache-Free Controls (HC), Migraine Patients Who Took Part as Healthy Controls (MH) or as Migraine Patients (MP) **eFigure 1.** Study Protocol **eTable 2.** Self-Reported Symptom Severity of Migraine for Headache-Free Controls (HC), Migraine Patients Who Took Part as Healthy Controls (MH) or as Migraine Patients (MP) **eFigure 2.** Results of Headache Questionnaires

Supplement 3.
Data Sharing Statement
