# Supplementary material for: Influence of Role Expectancy on Patient-Reported Outcomes Among Patients With Migraine: A Randomized Clinical Trial
Source: JAMA Netw Open. 2024 Apr 24;7(4):e243223. doi: 10.1001/jamanetworkopen.2024.3223 (PMC11043898; doi:10.1001/jamanetworkopen.2024.3223)
Supplement: Supplement 1. — Trial Protocol [file jamanetwopen-e243223-s001.pdf]

Universitätsklinikum Hamburg-Eppendorf

Institut für Systemische Neurowissenschaften

Prof. Dr. Christian Büchel
Direktor

Zentrum für Experimentelle Medizin
Haus W 34
Martinistraße 52
20246 Hamburg
Telefon: (040) 7410-59899
Telefax: (040) 7410-59955
sysneuro@uke.de
www.uke.de

Universitätsklinikum Hamburg-Eppendorf Martinistraße 52 20246 Hamburg
Institut für Systemische Neurowissenschaften

Ansprechpartner

Prof. Dr. A. May
Stellvertretender Institutsleiter
Leiter der Kopfschmerzambulanz
Tel: (040) 7410-59189
FAX: (040) 7410-59955
E-Mail: a.may@uke.de

Hamburg, 15.08. 2019

S t u d i e n p r o t o k o l l

„Visuelle Bewegungsstimulation bei Migränepatienten - eine fMRI-Studie“

“Visual motion stimulation in patients with migraine – A fMRI study”

(Bearb.-NR: PV7001, Version 3.0, 15.08.2019)

Persönliche Angaben

Namen und Dienststellungen des Leiters der Klinischen Prüfung (LKP)
(entspr. § 40 AMG oder § 17 MPG)
Prof. Dr. med. Arne May
Institut für systemische Neurowissenschaften
Universitäts-Krankenhaus Eppendorf,
Martinistr. 52, 20246 Hamburg
Tel. 040/7410-59189, FAX –59955
E-mail: a.may@uke.uni-hamburg.de

Mitarbeiter

Dr. Gabriela Ferreira Carvalho

Institut für systemische Neurowissenschaften

Zentrum für experimentelle Medizin

Martinistr. 52, 20246 Hamburg

E-Mail: gabriela.fisioterapia@gmail.com

Finanzierung der Studie (Sponsor)

Die Studie wird am NeuroImage Nord durchgeführt und ist somit über das Institut für Systemische Neurowissenschaften DFG- und BMBF-gefördert.

Abbreviations

BOLD: Blood oxygenation level dependent.

CoP: Center of pressure,

DHI: Dizziness handicap inventory,

fMRI: Functional magnetic resonance imaging,

ICHD-3: International classification of headache disorders,

MIDAS: Migraine disability assessment questionnaire,

MSSQ: Motion sickness susceptibility,

1. ABSTRACT

Background: Migraine is frequently associated with motion sickness, vestibular symptoms, and abnormal motion and visual processing. The triggered clinical symptoms and the brain mechanisms during self-motion visual stimulation were not yet investigated in this population.

Objective: To investigate the behavioral and brain responses from a visually simulated roller coaster ride of patients with migraine and headache-free controls.

Methods: Consecutive patients from a university-based hospital headache clinic (UKE) will be included, if eligible according to inclusion and exclusion criteria. A behavioral study will be conducted in order to assess the responses of 100 patients and 50 headache free controls to a self-motion stimulation paradigm based on a customized roller coaster video. Half of the patients with migraine will be informed that the study aims to investigate vestibular disorders, in order to consider a response bias in the analysis. A blinded examiner will give a questionnaire to the patient regarding features of migraine, vestibular symptoms, and motion sickness. Disability questionnaires related to migraine (MIDAS), dizziness (DHI) and motion sickness (MSSQ) will also be administered. During the video, quiet standing balance will be assessed on a foam surface using a camera and a marker positioned above the participant's head. Antero-posterior and medio-lateral displacement of the center of pressure will be measured. After watching the video, subjects will be asked about triggered symptoms and its duration. Subsequently, an additional fMRI study will be conducted with 20 patients with migraine and 20 controls in order to verify the brain responses to the paradigm. Blocks of roller coaster stimuli into several directions/velocities versus slow speed into a single direction will be randomly presented during a 30-minute period and displayed by means of Presentation 14.1 inside a Siemens Trio 3T scanner. Whole brain BOLD fMRI data will be acquired and processed with SPM12 and MATLAB. Groups will be compared for all outcomes with a significance level of 5% and a correlation between imaging and clinical features will also be assessed.

Expected results: The results of this project will elucidate the behavioral and brain responses of patients with migraine to self-movement simulated stimulus. We expect to find greater intolerance and associated symptoms to the stimulus in this group of patients, as well as a different pattern of brain activation, especially in regions such as the insula, cerebellum and brainstem. This outcome will be subject to context (response bias).

2. KEY-WORDS

Migraine Disorders, Headache, Dizziness, Visual Stimulation, Postural Balance, Functional Resonance Imaging.

3. BACKGROUND

Migraine is a chronic neurologic disorder characterized by episodic headache attacks with a worldwide prevalence ranging between 11% to 15%.[1, 2] It is ranked as the third most prevalent neurologic disorder and the first cause of years lived with disability in individuals under 50 years of age.[3, 4] Further than headache, patients with migraine may present gastrointestinal disorders, several neurologic and autonomic alterations, among many other comorbidities.[5, 6]

It has been verified that this population has an increased prevalence of symptoms related to visual and motion perception, including motion sickness and vestibular symptoms.[7-15] Motion sickness (nausea) is indeed considered part of the diagnostic criteria for migraine according to the International Classification of Headache Disorders (ICHD-3).[16] In addition, dizziness can be considered inherent of the migraine condition,[7, 8] along with other common symptoms such as cutaneous allodynia,[17] photo and phonophobia.[18]

Migraineurs exhibit functional movement alterations from an early age[19] with a delayed development and integration impairment of the visual motion processing.[20, 21] Under visual motion stimuli, this anomaly in the motion/orientation perception[22-27] and processing pathways is still observed in adults[25-30] and can induce balance instability.[31, 32]

Impairment of balance control is a common sign related to motion perception alterations and vestibular symptoms. It was also already verified that patients with migraine exhibit balance control changes in conditions of simple quiet standing[7, 33-42] and during functional activities such as walking, transposing obstacles or sit to standing tasks.[37, 41] In the presence of migraine aura or migraine chronicity, differences compared to healthy controls are more pronounced, especially regarding the self-report of falls[43] and quiet standing balance tasks.[38, 39]

All of these signs and symptoms can arise from a sensory information mismatch among the proprioceptive, the vestibular and visual systems.[44-46] For example, in the context of illusory sensation of self-movements when watching a visual motion stimulus, the visual afferences does not match the vestibular or proprioceptive cues, which is predicted based on previous experiences.[47]

The divergence between expected and perceived sensorial information can be triggered by underling disturbances at a peripheral level, including the proprioceptive, exteroceptive, labyrinthic and visual afferents; and/or in structures of central nervous system, such as the inner ear, brain stem, cerebellum, basal ganglia and cortical hemispheres.[48, 49]

The mechanism of migraine susceptibility to these symptoms is still not well understood.[10, 50] Possibilities include brain hyperexcitability[15, 22, 26, 29, 51, 52] and lack of habituation,[26, 53] overlap between trigeminal and vestibular pathways,[41, 42, 54-56] structural and functional abnormality of the visual processing network,[26, 57, 58] altered function of the inner ear[33, 36, 42, 59, 60] and vasospasm-induced ischemia of the labyrinth and brainstem.[61, 62]

Some studies additionally demonstrate that motion intolerance, vestibular symptoms and abnormalities in eye movements can be related to the known impairment of the cerebellar function and its structural changes in migraine patients.[13-15, 31, 63-70] Indeed, extensive sensory input from the vestibular,[71] proprioceptive and visual systems converge into the cerebellum[72] and into the brainstem.[73] Moreover, the cerebellum plays a role in the control of somatic and autonomic responses to self-movement and moving environments,[73, 74] process information regarding velocity proprieties, spatial orientation[75-78] and navigation,[79] linear acceleration and gravity.[80-83]

Moving dots, black and white checkerboards, rings and gratings, among other types of visual stimuli, have been widely used to stimulate migraine patients, with the aim to study the motion/visual processing and the pathophysiological mechanisms related to this disease. Visual stimulation can activate the nociceptive trigeminal nucleus in chronic migraine[84] and trigger associated symptoms such as nausea, photophobia, tenderness and pain sensitivity.[11] Furthermore, trigeminal stimulation can induce vestibular asymmetry in the headache-free interval.[85]

However, the brain processing mechanisms through functional magnetic resonance imaging (fMRI) related to the self-motion under gravity conditions have not been investigated in migraineurs so far. Visual self-motion stimuli, based mainly on roller coaster simulated rides, have been used to study the motion and gravity processing,[86] motion sickness,[87-89] and brain physiologic mechanisms,[90-94] in both healthy subjects[86-89, 92-94] and patients with vestibular disorders.[90] Vertical motion stimulus can

induce activations in the insula,[90, 92, 94] the parieto-insular vestibular cortex,[93] thalamus, pre-frontal areas and the cerebellum;[94] while horizontal motion activate mainly the hippocampus and parahippocampal regions.[94]

Taking all these previous described evidence together, we hypothesize that patients with migraine would exhibit differences compared to headache-free subjects regarding clinical symptoms and also brain processing mechanisms during visual self-motion stimulation by means of roller coaster simulation rides. We also suggest that the heterogeneity of migraine presentation considering the presence/absence of vestibular symptoms, presence of aura and/or attacks frequency would influence the behavior and brain responses to the stimuli.

Significance

The clinical symptoms and the brain mechanisms triggered by self-motion stimulation were not yet investigated in patients with migraine. Since those patients exhibit substantial alterations in the cerebellum, impairment of visual, vestibular and motion processing and also self-report of motion sickness susceptibility, balance changes and vestibular symptoms; the study of the mechanisms under gravity simulation is pertinent. The understanding of the brain and cerebellar activity under simulated roller coaster rides would contribute to the knowledge of migraine pathophysiologic mechanisms and interactions with established disease comorbidities.

Furthermore, it would allow the identification of common or distinct brain functioning among patients considering the differences in the migraine spectrum, including attack frequency, presence/absence of aura and self-report of motion sickness or vestibular symptoms. The identification of differences among patients' presentation is of importance due to its potential influence on the assessment, treatment plan, and prognosis. It would improve the recognition of different levels of disability and may make feasible the inclusion of a tailored rationale of treatment in the clinical practice.

4. OBJECTIVES

This present study aims to investigate the clinical responses and also the brain activity of

patients with migraine during visual self-movement stimulation.

5. METHODS

This cross-sectional study will be conducted at the headache clinic of the University Medical Center Hamburg-Eppendorf, Germany. Patients with migraine will be identified by neurologists with expertise in headaches and diagnosed according to the ICHD-III.[16] Two experiments will be performed in order to address the study objectives.

All participants will sign the study consent form before enrollment. Once enrolled, the subjects will have the right to pause or terminate his/her participation at any time during the study.

5.1. Behavioral study – Experiment I

In order to test the patients' response to the roller coaster paradigm, initially they will be invited to watch a roller coaster video and clinical measurements will be recorded.

Sample

The study enrollment consists initially of 100 consecutive subjects with migraine and 50 controls. After a pilot phase, a sample size calculation will be performed, and the number of participants will be redefined in order to ensure a power of 80% and at least moderate effect size (>0.5). In order to verify the effect of response bias, part of the patients with migraine will be informed that the study aims to investigate vestibular disorders instead of headache disorders.

Eligible patients according to the inclusion and exclusion criteria (Table 1) will be invited to participate.

Table 1. Enrollment criteria of the experiment I.

Inclusion criteria:

1. Males and females with age over 18,
2. Written consent to the investigation

Exclusion Criteria:

1. Migraine attack on the day of the appointment,
2. History of any neurological disease,
3. No other relevant headache diagnosis other than migraine (e.g., medication overuse headache).
4. Self-report of diagnosis of vestibular disease,
5. History of trauma or pathology of the cervical spine (e.g., whiplash associated disorder),
6. Pregnancy,
7. Other relevant musculoskeletal, systemic or psychiatric disease.

Study Procedures

A. Roller Coaster Paradigm

A roller coaster ride was simulated by displaying a first-person perspective view of forward self-motion (Fig.1) in a full HD monitor (23.8", EIZO, California, EUA). The custom roller-coaster used in this study was created using the *No Limits Coaster* software (www.nolimitscoaster.com, Mad Data, Joerg Henseler, Erkrath, Germany). The ride simulation consists of animated scenes with tracks in the up, down, left and right direction, with horizontal and vertical perspectives. The acceleration and movement velocity varied according to the ride direction.

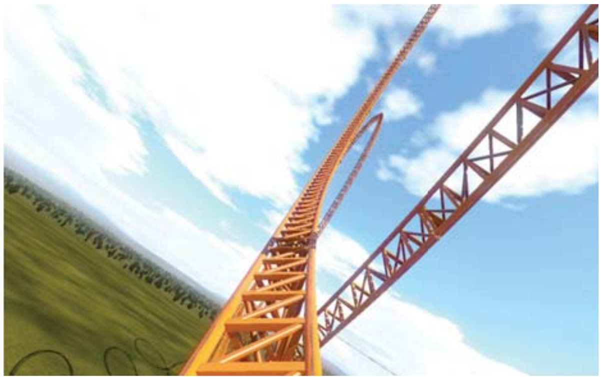

Figure 1. Example of patients' screen view of the roller coaster paradigm. It is simulated as if the viewer were a passenger sitting in the front-car and looking straight ahead.

B. Clinical assessment

An assessor blinded to the patient group allocation will carry out all the assessment procedures.

After the physician consultation, the eligible patients that agreed to participate in the study will receive a set of questionnaires with inquiries regarding demographical and headache features including age, occupation, migraine onset, intensity, frequency and migraine disability (migraine disability assessment questionnaire – MIDAS).[95] Furthermore, they will complete the PHQ-8 questionnaire in order to screen for presence of depression.

The presence of vestibular symptoms will also be assessed. Patients will be encouraged to describe their symptoms according to the Barany's Classification of Vestibular Disorders[96] including its form (internal/external vertigo, dizziness or postural symptoms), its duration, frequency and concomitant occurrence with migraine attacks. Motion sickness susceptibility with the MSSQ – short version questionnaire[97] and the disability level related to vestibular symptoms with the Dizziness Handicap Inventory (DHI-G) will be assessed.[98] A staff member will be available at all times during the data collection in order to solve any possible query.

After the interview procedure, patients will be exposed to the roller coaster paradigm. They will be asked to stay in a sitting position resting their faces in a chin-head rest and concentrate in the screen positioned 25 cm from the head, in the eye level. The duration of the video is 5 minutes and patients will be asked about triggered symptoms following the video (i.e. motion sickness, dizziness) and its duration will be recorded.

5.2. fMRI Study – Experiment II

Once it was verified that the virtual roller coaster stimulation can trigger vestibular symptoms in migraine patients, an fMRI assessment will be carried out in order to understand the brain responses to the visual self-motion stimuli.

Sample

Twenty patients with migraine and twenty controls will be invited to participate in the study according to the following inclusion/exclusion criteria (table 2).

Table 2. Enrollment criteria of the experiment II.

***Inclusion criteria*:**

1. Males and females with age over 18,
2. Sufficient cooperation of the patient and ability to lie quietly on the back for 45-60 minutes.
3. Written consent to the investigation

Exclusion Criteria:

1. Migraine attack on the day of the appointment,
2. History of any neurological disease,
3. No other relevant headache diagnosis other than migraine (e.g., medication overuse headache).
4. Self-report of diagnosis of vestibular disease,
5. History of trauma or pathology of the cervical spine (e.g., whiplash associated disorder),
6. Other relevant musculoskeletal, systemic or psychiatric disease.
7. Pregnancy,
8. Smoking,
9. History of claustrophobia,
10. Any other general contraindications for fMRI exam (e.g. pacemakers, incompatible metallic implants).

Study Procedures

The same paradigm described above for the behavioral study will be displayed in AVI videos using Presentation 14.1 (Neurobehavioral Systems Inc., Albany, Canada), at 1024×768 pixels, 60 frames per second, 24°×19° visual angle. Blocks of roller coaster stimuli motion (30sec.) will be inter-leaved with low speed roller coaster motion into a single direction (15sec. duration) for 30 minutes. The order of the roller coaster direction component was randomly changed in each block in order to avoid habituation during fMRI scanning.

A. fMRI acquisition

The image scanning will be performed in a Siemens Trio 3T scanner (Siemens, Erlangen, Germany) with a 32-channel head coil. Whole brain BOLD fMRI data acquisition will use an echo planar imaging sequence (repetition time 2.62 s, echo time 30 ms, flip angle 80°, field of view 220mm2, acceleration mode GRAPPA, spatial resolution 2.0x2.0x2.0 mm, acquisition matrix 110x110x40), covering the sTN in the brainstem, the cerebellum, the midbrain, insula and most of the cortex. Each volume will consist of 40 axial slices (slice thickness 2 mm, gap 1 mm).

B. Image processing

Functional images will be analyzed using SPM12 and MATLAB (The MathWorks Inc., Natick, MA). During preprocessing, images will be imported and converted to 4D nifti files, denoised via the spatially adaptive nonlocal means algorithm as implemented in the CAT12 toolbox, slice time corrected, realigned, normalized into Montreal Neurological Institute space via a segmentation-normalization sequence, upsampled to a spatial resolution of 1 × 1 × 1 mm3, and smoothed using a 4-mm full width at half maximum isotropic Gaussian kernel. The highpass filters' cutoff frequency will be set to 128 sec.

For each participant, general linear models (GLM) will be used to assess regionally specific effects of stimuli parameters on BOLD activations. Trials will be modeled as epochs of variable duration and convolved with the SPM8 hemodynamic response function. First-level GLMs included motion stimuli in several directions *versus* slow motion in one direction conditions.

6. DATA ANALYSIS

Initially, descriptive statistics will be used: absolute and relative frequencies according to the variables characteristics. Continuous variables will be summarized using averages and standard deviations (if normally distributed according to the Kolmogorov-Smirnov or the Shapiro-Wilk test) and median and range if nonparametrically distributed.

Differences between the migraine and control groups will be determined by an independent two-way Student's T-test, or Mann-Whitney U test according to the distribution of the variables for continuous variables balance outcomes, migraine features and demographical data. Categorical variables will be studied using the chi-square or Fisher's exact test.

Differences in brain activity between controls and migraineurs will calculated by independent two-way Student's T-test with an uncorrected threshold of $p<0.001$. For each contrast of interest, regression coefficients will be calculated between brain activity and clinical features of migraine, DHI-G scores, Midas and MSSQ within the migraine group to test the hypothesis that disease severity modulates brain responses. The significance level will be set at 0.05 and p-value, and 95% confidence intervals will be reported.

7. EXPECTED RESULTS

The results of this project will elucidate the behavioral and brain responses of patients with migraine to self-movement simulated stimulus. We expect to find greater intolerance and associated symptoms to the stimulus in this group of patients, as well as, a different pattern of brain activation, especially in regions such as insula, cerebellum and brainstem.

8. WORK PLAN AND ACTIVITIES TIMETABLE

**Stages**	**Study Period (months from September 2019 to April 2021)**										
	**1**	**2**	**3**	**4**	**5**	**6**	**7**	**8**	**9**	**10**	**11**
Project approval from the local Ethics Committee	X										
Sample Selection		X	X	X	X	X	X	X			
Experiment I data Collection		X	X	X	X	X					
Experiment II data Collection							X	X			
Data Analysis								X	X		
Bibliographic review								X	X	X	
Manuscripts preparation and submission										X	X

9. REFERENCES

1. Stovner LJ, Andree C. Prevalence of headache in Europe: a review for the Eurolight project. J Headache Pain 2010;11:289-299.

2. Lipton RB, Bigal ME, Diamond M, Freitag F, Reed ML, Stewart WF. Migraine prevalence, disease burden, and the need for preventive therapy. Neurology 2007;68:343-349.

3. Global Burden of Disease Study C. Global, regional, and national incidence, prevalence, and years lived with disability for 301 acute and chronic diseases and injuries in 188 countries, 1990-2013: a systematic analysis for the Global Burden of Disease Study 2013. Lancet 2015;386:743-800.

4. Steiner TJ, Stovner LJ, Vos T, Jensen R, Katsarava Z. Migraine is first cause of disability in under 50s: will health politicians now take notice? J Headache Pain 2018;19:17.

5. Goadsby PJ, Lipton RB, Ferrari MD. Migraine--current understanding and treatment. N Engl J Med 2002;346:257-270.

6. Silberstein SD. Migraine. Lancet 2004;363:381-391.

7. Calhoun AH, Ford S, Pruitt AP, Fisher KG. The point prevalence of dizziness or vertigo in migraine and factors that influence presentation. Headache 2011;51:1388-1392.

8. Carvalho GF, Vianna-Bell FH, Florencio LL, et al. Presence of vestibular symptoms and related disability in migraine with and without aura and chronic migraine. Cephalalgia 2018:333102418769948.

9. Furman JM, Marcus DA. Migraine and motion sensitivity. Continuum (Minneap Minn) 2012;18:1102-1117.

10. Drummond PD. Triggers of motion sickness in migraine sufferers. Headache 2005;45:653-656.

11. Drummond PD. Motion sickness and migraine: optokinetic stimulation increases scalp tenderness, pain sensitivity in the fingers and photophobia. Cephalalgia 2002;22:117-124.

12. Drummond PD, Granston A. Facial pain increases nausea and headache during motion sickness in migraine sufferers. Brain 2004;127:526-534.

13. Murdin L, Chamberlain F, Cheema S, et al. Motion sickness in migraine and vestibular disorders. J Neurol Neurosurg Psychiatry 2015;86:585-587.

14. Jeong SH, Oh SY, Kim HJ, Koo JW, Kim JS. Vestibular dysfunction in migraine: effects of associated vertigo and motion sickness. J Neurol 2010;257:905-912.

15. Boldingh MI, Ljostad U, Mygland A, Monstad P. Vestibular sensitivity in vestibular migraine: VEMPs and motion sickness susceptibility. Cephalalgia 2011;31:1211-1219.

16. Headache Classification Committee of the International Headache Society (IHS) The International Classification of Headache Disorders, 3rd edition. Cephalalgia 2018;38:1-211.

17. Lovati C, D'Amico D, Bertora P, et al. Acute and interictal allodynia in patients with different headache forms: an Italian pilot study. Headache 2008;48:272-277.

18. Main A, Dowson A, Gross M. Photophobia and phonophobia in migraineurs between attacks. Headache 1997;37:492-495.

19. Youssef PE, Mack KJ. Abnormal movements in children with migraine. Journal of child neurology 2015;30:285-288.

20. Precenzano F, Ruberto M, Parisi L, et al. Visual-spatial training efficacy in children affected by migraine without aura: a multicenter study. Neuropsychiatr Dis Treat 2017;13:253-258.

21. Braunitzer G, Rokszin A, Kobor J, Benedek G, Nagy A, Kincses ZT. Delayed development of visual motion processing in childhood migraine. Cephalalgia 2012;32:492-496.

22. Antal A, Temme J, Nitsche MA, Varga ET, Lang N, Paulus W. Altered motion perception in migraineurs: evidence for interictal cortical hyperexcitability. Cephalalgia 2005;25:788-794.

23. Miller MA, O'Leary CJ, Allen PD, Crane BT. Human Vection Perception Using Inertial Nulling and Certainty Estimation: The Effect of Migraine History. PLoS One 2015;10:e0135335.

24. McKendrick AM, Badcock DR, Gurgone M. Vernier acuity is normal in migraine, whereas global form and global motion perception are not. Investigative ophthalmology & visual science 2006;47:3213-3219.

25. McKendrick AM, Badcock DR. Motion processing deficits in migraine. Cephalalgia 2004;24:363-372.

26. O'Hare L, Hibbard PB. Visual processing in migraine. Cephalalgia 2016;36:1057-1076.

27. Shepherd AJ, Beaumont HM, Hine TJ. Motion processing deficits in migraine are related to contrast sensitivity. Cephalalgia 2012;32:554-570.

28. Singh P, Shepherd AJ. Enhanced Motion Aftereffects in Migraine Are Related to Contrast Sensitivity: Implications for Models of Differences in Precortical/Cortical Function. Investigative ophthalmology & visual science 2016;57:1228-1234.

29. Griebe M, Flux F, Wolf ME, Hennerici MG, Szabo K. Multimodal assessment of optokinetic visual stimulation response in migraine with aura. Headache 2014;54:131-141.

30. Ditchfield JA, McKendrick AM, Badcock DR. Processing of global form and motion in migraineurs. Vision Res 2006;46:141-148.

31. Lim YH, Kim JS, Lee HW, Kim SH. Postural Instability Induced by Visual Motion Stimuli in Patients With Vestibular Migraine. Frontiers in neurology 2018;9:433.

32. Imaizumi S, Honma M, Hibino H, Koyama S. Illusory visual motion stimulus elicits postural sway in migraine patients. Frontiers in psychology 2015;6:542.

33. Celebisoy N, Gokcay F, Sirin H, Bicak N. Migrainous vertigo: clinical, oculographic and posturographic findings. Cephalalgia 2008;28:72-77.

34. Ongun N, Atalay NS, Degirmenci E, Sahin F, Bir LS. Tetra-ataxiometric Posturography in Patients with Migrainous Vertigo. Pain physician 2016;19:E87-96.

35. Panichi R, Cipriani L, Sarchielli P, et al. Balance control impairment induced after OKS in patients with vestibular migraine: an intercritical marker. European archives of oto-rhino-laryngology : official journal of the European Federation of Oto-Rhino-Laryngological Societies 2015;272:2275-2282.

36. Teggi R, Colombo B, Bernasconi L, Bellini C, Comi G, Bussi M. Migrainous vertigo: results of caloric testing and stabilometric findings. Headache 2009;49:435-444.

37. Carvalho GF, Florencio LL, Pinheiro CF, Dach F, Bigal ME, Bevilaqua-Grossi D. Functional Balance Deterioration on Daily Activities in Patients With Migraine: A Controlled Study. Am J Phys Med Rehabil 2017;97:90-95.

38. Carvalho GF, Bonato P, Florencio LL, et al. Balance Impairments in Different Subgroups of Patients With Migraine. Headache 2017;57:363-374.

39. Carvalho GF, Chaves TC, Dach F, et al. Influence of Migraine and of Migraine Aura on Balance and Mobility - A Controlled Study. Headache 2013;53:1116-1122.

40. Akdal G, Balci BD, Angin S, Ozturk V, Halmagyi GM. A longitudinal study of balance in migraineurs. Acta Otolaryngol 2012;132:27-32.

41. Akdal G, Donmez B, Ozturk V, Angin S. Is balance normal in migraineurs without history of vertigo? Headache 2009;49:419-425.

42. Baker BJ, Curtis A, Trueblood P, Vangsnes E. Vestibular functioning and migraine: comparing those with and without vertigo to a normal population. J Laryngol Otol 2013;127:1169-1176.

43. Carvalho GF, Almeida CS, Florencio LL, et al. Do patients with migraine experience an increased prevalence of falls and fear of falling? A cross-sectional study. Physiotherapy 2018.

44. Treleaven J. Sensorimotor disturbances in neck disorders affecting postural stability, head and eye movement control. Manual therapy 2008;13:2-11.

45. Bles W, Bos JE, Kruit H. Motion sickness. Curr Opin Neurol 2000;13:19-25.

46. Bertolini G, Straumann D. Moving in a Moving World: A Review on Vestibular Motion Sickness. Frontiers in neurology 2016;7:14.

47. Bles W, Bos JE, de Graaf B, Groen E, Wertheim AH. Motion sickness: only one provocative conflict? Brain Res Bull 1998;47:481-487.

48. Cronin T, Arshad Q, Seemungal BM. Vestibular Deficits in Neurodegenerative Disorders: Balance, Dizziness, and Spatial Disorientation. Frontiers in neurology 2017;8:538.

49. Bronstein AM. Multisensory integration in balance control. Handb Clin Neurol 2016;137:57-66.

50. Casani AP, Sellari-Franceschini S, Napolitano A, Muscatello L, Dallan I. Otoneurologic dysfunctions in migraine patients with or without vertigo. Otol Neurotol 2009;30:961-967.

51. Huang J, Cooper TG, Satana B, Kaufman DI, Cao Y. Visual distortion provoked by a stimulus in migraine associated with hyperneuronal activity. Headache 2003;43:664-671.

52. Rogalinski S, Rambold HA. Probing Early Motion Processing With Eye Movements: Differences of Vestibular Migraine, Migraine With and Without Aura in the Attack Free Interval. Headache 2018;58:275-286.

53. Roceanu A, Allena M, De Pasqua V, Bisdorff A, Schoenen J. Abnormalities of the vestibulocollic reflex are similar in migraineurs with and without vertigo. Cephalalgia 2008;28:988-990.

54. Furman JM, Marcus DA, Balaban CD. Vestibular migraine: clinical aspects and pathophysiology. Lancet Neurol 2013;12:706-715.

55. Furman JM, Sparto PJ, Soso M, Marcus D. Vestibular function in migraine-related dizziness: a pilot study. J Vestib Res 2005;15:327-332.

56. Ishizaki K, Mori N, Takeshima T, et al. Static stabilometry in patients with migraine and tension-type headache during a headache-free period. Psychiatry Clin Neurosci 2002;56:85-90.

57. Granziera C, DaSilva AF, Snyder J, Tuch DS, Hadjikhani N. Anatomical alterations of the visual motion processing network in migraine with and without aura. PLoS Med 2006;3:e402.

58. Luedtke K, Schulte LH, May A. Visual processing in migraineurs depends on the migraine cycle. Ann Neurol 2019;85:280-283.

59. Boldingh MI, Ljostad U, Mygland A, Monstad P. Comparison of Interictal Vestibular Function in Vestibular Migraine vs Migraine Without Vertigo. Headache 2013;53:1123-1133.

60. Yollu U, Uluduz DU, Yilmaz M, et al. Vestibular migraine screening in a migraine-diagnosed patient population, and assessment of vestibulocochlear function. Clin Otolaryngol 2016.

61. Hong SM, Kim SK, Park CH, Lee JH. Vestibular-evoked myogenic potentials in migrainous vertigo. Otolaryngol Head Neck Surg 2011;144:284-287.

62. Parker W. Migraine and the vestibular system in adults. Am J Otol 1991;12:25-34.

63. Wang J, Lewis RF. Contribution of intravestibular sensory conflict to motion sickness and dizziness in migraine disorders. J Neurophysiol 2016;116:1586-1591.

64. Dai M, Raphan T, Cohen B. Labyrinthine lesions and motion sickness susceptibility. Exp Brain Res 2007;178:477-487.

65. Harno H, Hirvonen T, Kaunisto MA, et al. Subclinical vestibulocerebellar dysfunction in migraine with and without aura. Neurology 2003;61:1748-1752.

66. Milhaud D, Bogousslavsky J, van Melle G, Liot P. Ischemic stroke and active migraine. Neurology 2001;57:1805-1811.

67. Hoekstra-van Dalen RA, Cillessen JP, Kappelle LJ, van Gijn J. Cerebral infarcts associated with migraine: clinical features, risk factors and follow-up. J Neurol 1996;243:511-515.

68. Vincent M, Hadjikhani N. The cerebellum and migraine. Headache 2007;47:820-833.

69. Mehnert J, May A. Functional and structural alterations in the migraine cerebellum. J Cereb Blood Flow Metab 2017:271678X17722109.

70. Kruit MC, van Buchem MA, Hofman PA, et al. Migraine as a risk factor for subclinical brain lesions. JAMA 2004;291:427-434.

71. Angelaki DE, Hess BJ. Inertial representation of angular motion in the vestibular system of rhesus monkeys. I. Vestibuloocular reflex. J Neurophysiol 1994;71:1222-1249.

72. Barmack NH, Shojaku H. Vestibular and visual climbing fiber signals evoked in the uvula-nodulus of the rabbit cerebellum by natural stimulation. J Neurophysiol 1995;74:2573-2589.

73. Marcus DA, Furman JM, Balaban CD. Motion sickness in migraine sufferers. Expert Opin Pharmacother 2005;6:2691-2697.

74. Balaban CD. Vestibular autonomic regulation (including motion sickness and the mechanism of vomiting). Curr Opin Neurol 1999;12:29-33.

75. Cohen B, John P, Yakushin SB, Buettner-Ennever J, Raphan T. The nodulus and uvula: source of cerebellar control of spatial orientation of the angular vestibulo-ocular reflex. Ann N Y Acad Sci 2002;978:28-45.

76. Solomon D, Cohen B. Stimulation of the nodulus and uvula discharges velocity storage in the vestibulo-ocular reflex. Exp Brain Res 1994;102:57-68.

77. Sheliga BM, Yakushin SB, Silvers A, Raphan T, Cohen B. Control of spatial orientation of the angular vestibulo-ocular reflex by the nodulus and uvula of the vestibulocerebellum. Ann N Y Acad Sci 1999;871:94-122.

78. Laurens J, Angelaki DE. The functional significance of velocity storage and its dependence on gravity. Exp Brain Res 2011;210:407-422.

79. Igloi K, Doeller CF, Paradis AL, et al. Interaction Between Hippocampus and Cerebellum Crus I in Sequence-Based but not Place-Based Navigation. Cereb Cortex 2015;25:4146-4154.

80. Laurens J, Meng H, Angelaki DE. Computation of linear acceleration through an internal model in the macaque cerebellum. Nat Neurosci 2013;16:1701-1708.

81. Rosenberg A, Angelaki DE. Gravity influences the visual representation of object tilt in parietal cortex. J Neurosci 2014;34:14170-14180.

82. Yakusheva TA, Blazquez PM, Chen A, Angelaki DE. Spatiotemporal properties of optic flow and vestibular tuning in the cerebellar nodulus and uvula. J Neurosci 2013;33:15145-15160.

83. Indovina I, Maffei V, Bosco G, Zago M, Macaluso E, Lacquaniti F. Representation of visual gravitational motion in the human vestibular cortex. Science 2005;308:416-419.

84. Schulte LH, Allers A, May A. Visual stimulation leads to activation of the nociceptive trigeminal nucleus in chronic migraine. Neurology 2018;90:e1973-e1978.

85. Marano E, Marcelli V, Di Stasio E, et al. Trigeminal stimulation elicits a peripheral vestibular imbalance in migraine patients. Headache 2005;45:325-331.

86. Indovina I, Mazzarella E, Maffei V, Cesqui B, Passamonti L, Lacquaniti F. Sound-evoked vestibular stimulation affects the anticipation of gravity effects during visual self-motion. Exp Brain Res 2015;233:2365-2371.

87. Nalivaiko E, Davis SL, Blackmore KL, Vakulin A, Nesbitt KV. Cybersickness provoked by head-mounted display affects cutaneous vascular tone, heart rate and reaction time. Physiology & behavior 2015;151:583-590.

88. Gavgani AM, Nesbitt KV, Blackmore KL, Nalivaiko E. Profiling subjective symptoms and autonomic changes associated with cybersickness. Auton Neurosci 2017;203:41-50.

89. Mazloumi Gavgani A, Hodgson DM, Nalivaiko E. Effects of visual flow direction on signs and symptoms of cybersickness. PLoS One 2017;12:e0182790.

90. Riccelli R, Passamonti L, Toschi N, et al. Altered Insular and Occipital Responses to Simulated Vertical Self-Motion in Patients with Persistent Postural-Perceptual Dizziness. Frontiers in neurology 2017;8:529.

91. Jancke L, Cheetham M, Baumgartner T. Virtual reality and the role of the prefrontal cortex in adults and children. Front Neurosci 2009;3:52-59.

92. Li D, Zucker NL, Kragel PA, Covington VE, LaBar KS. Adolescent development of insula-dependent interoceptive regulation. Dev Sci 2017;20.

93. Riccelli R, Indovina I, Staab JP, et al. Neuroticism modulates brain visuo-vestibular and anxiety systems during a virtual rollercoaster task. Hum Brain Mapp 2017;38:715-726.

94. Indovina I, Maffei V, Pauwels K, Macaluso E, Orban GA, Lacquaniti F. Simulated self-motion in a visual gravity field: sensitivity to vertical and horizontal heading in the human brain. Neuroimage 2013;71:114-124.

95. Stewart WF, Lipton RB, Dowson AJ, Sawyer J. Development and testing of the Migraine Disability Assessment (MIDAS) Questionnaire to assess headache-related disability. Neurology 2001;56:S20-28.

96. Bisdorff AR, Staab JP, Newman-Toker DE. Overview of the International Classification of Vestibular Disorders. Neurol Clin 2015;33:541-550, vii.

97. Golding JF. Motion sickness susceptibility questionnaire revised and its relationship to other forms of sickness. Brain Res Bull 1998;47:507-516.

98. Kurre A, van Gool CJ, Bastiaenen CH, Gloor-Juzi T, Straumann D, de Bruin ED. Translation, cross-cultural adaptation and reliability of the german version of the dizziness handicap inventory. Otol Neurotol 2009;30:359-367.
