# Supplementary material for: Influence of Role Expectancy on Patient-Reported Outcomes Among Patients With Migraine: A Randomized Clinical Trial
Source: JAMA Netw Open. 2024 Apr 24;7(4):e243223. doi: 10.1001/jamanetworkopen.2024.3223 (PMC11043898; doi:10.1001/jamanetworkopen.2024.3223)
Supplement: Supplement 2. — eTable 1. Ratings of the Visual Intervention (Roller Coaster Video) for Headache-Free Controls (HC), Migraine Patients Who Took Part as Healthy Controls (MH) or as Migraine Patients (MP) eFigure 1. Study Protocol eTable 2. Self-Reported Symptom Severity of Migraine for Headache-Free Controls (HC), Migraine Patients Who Took Part as Healthy Controls (MH) or as Migraine Patients (MP) eFigure 2. Results of Headache Questionnaires [file jamanetwopen-e243223-s002.pdf]

Supplemental Online Content

May A, Carvalho GF, Schwarz A, Basedau H. Influence of role expectancy on patient-reported outcomes among patients with migraine. *JAMA Netw Open.* 2024;7(3):e243223. doi:10.1001/jamanetworkopen.2024.3223

eTable 1. Ratings of the Visual Intervention (Roller Coaster Video) for Headache-Free Controls (HC), Migraine Patients Who Took Part as Healthy Controls (MH) or as Migraine Patients (MP)
eFigure 1. Study Protocol
eTable 2. Self-Reported Symptom Severity of Migraine for Headache-Free Controls (HC), Migraine Patients Who Took Part as Healthy Controls (MH) or as Migraine Patients (MP).

eFigure 2. Results of Headache Questionnaires

This supplemental material has been provided by the authors to give readers additional information about their work.

© 2024 May A et al. *JAMA Network Open.*

Rating of Visual intervention	***HC***	***MH***	***MP***	***p-value***
Video 1: **Simulator Sickness Questionnaire** (SSQ) total score median [IQR]	7.48 [11.22]	14.96 [18.7]	26.18 [37.4]	[b]p<.001
Video 2: **Simulator Sickness Questionnaire** (SSQ) total score median [IQR]	7.48 [18.7]	14.96 [26.18]	29.92 [49.56]	[b]p<.001

eTable 1: Ratings of the visual intervention (roller coaster video) for headache-free controls (HC), migraine patients who took part as healthy controls (MH) or as migraine patients (MP). Tested by [b]Kruskal-Wallis test.

self reported symptom severity	***HC***	***MH***	***MP***	***p-value***
Self-reported monthly Headache frequency, median [IQR], days/month		5 [8]	7 [11.5]	[c]p=.008
Migraine Disability Assessment (MiDAS), score median [IQR]		24.75 [35.75]	35 [43]	[c]p=.005

eTable 2: Self-reported symptom severity of migraine for headache-free controls (HC), migraine patients who took part as healthy controls (MH) or as migraine patients (MP). Tested by [c]Mann-Whitney-U test

© 2024 May A et al. *JAMA Network Open.*

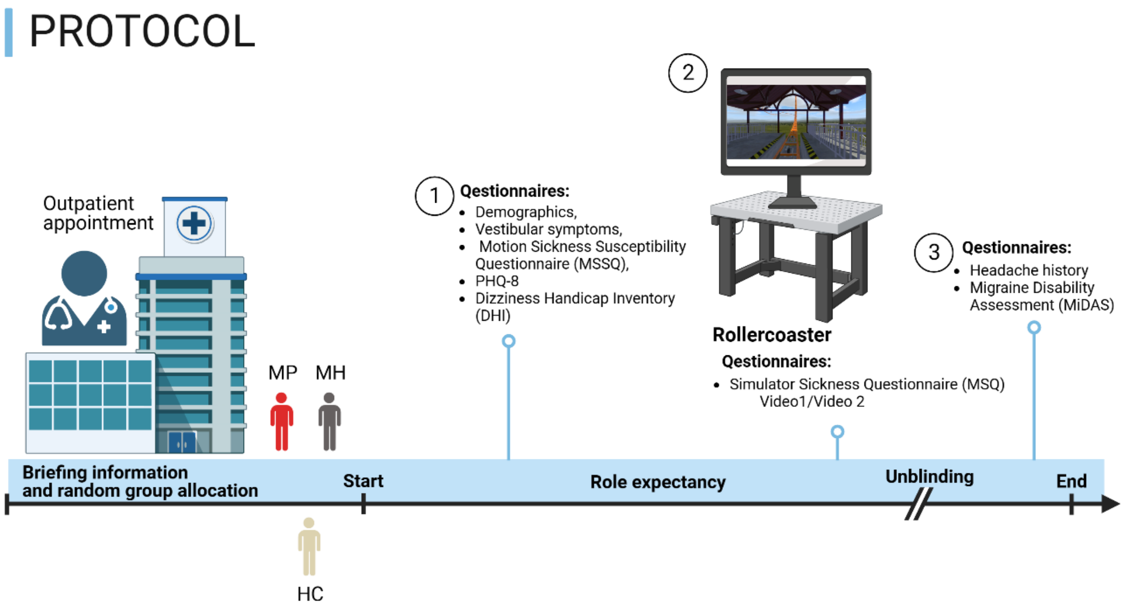

eFigure 1: Study protocol

Recruitment of patients with migraine through the outpatient clinic and assignment of the context to each patient presenting as patient with migraine being patient [MP] or patient with migraine being healthy control [MH]. All participants filled out demographic questionnaires (1) visual stimulation using a roller coaster video and vestibular symptoms questionnaires (2), and finally headache questionnaires (3).

© 2024 May A et al. *JAMA Network Open.*

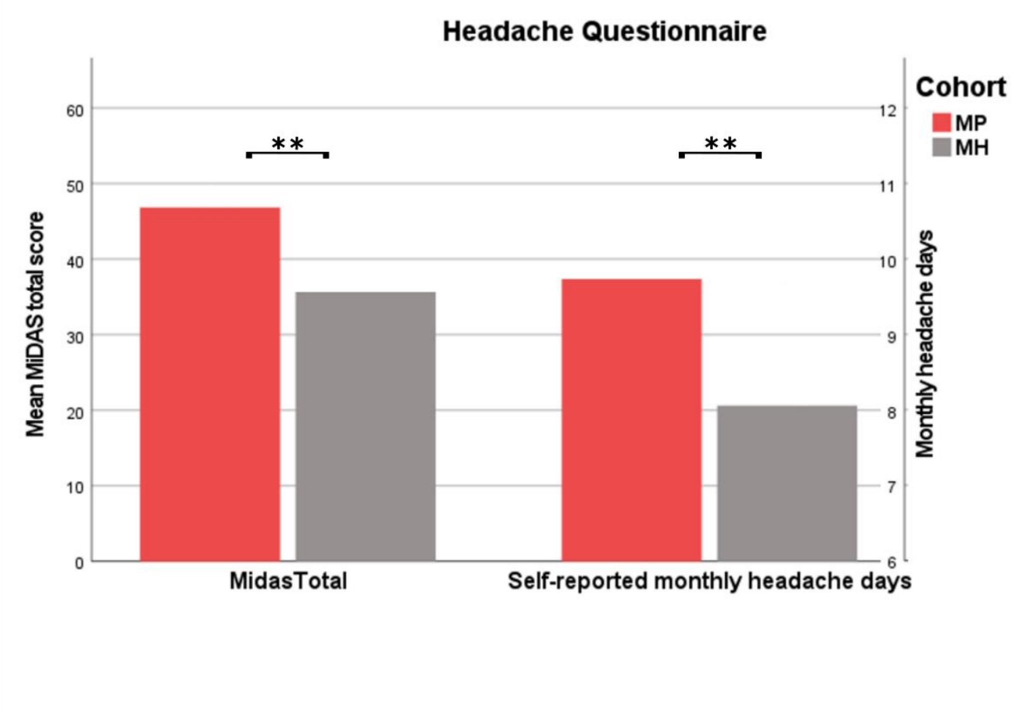

eFigure 2: Results of headache questionnaires

Barplots showing the mean of the total score in the Migraine Disability Assessment (left) and the self-reported monthly headache days (right). Patients with migraine recruited as patients [MP] in red, and patients with migraine recruited as healthy control [MH] in grey. Significant comparisons are labelled p <0.01 with **.

© 2024 May A et al. *JAMA Network Open.*
