# Supplementary material for: Influence of Role Expectancy on Patient-Reported Outcomes Among Patients With Migraine: A Randomized Clinical Trial
Source: JAMA Netw Open. 2024 Apr 24;7(4):e243223. doi: 10.1001/jamanetworkopen.2024.3223 (PMC11043898; doi:10.1001/jamanetworkopen.2024.3223)
Supplement: Supplement 3. — Data Sharing Statement [file jamanetwopen-e243223-s003.pdf]

Data Sharing Statement

May. Influence of Role Expectancy on Patient-Reported Outcomes Among Patients With Migraine. *JAMA Netw Open*. Published March 27, 2024.
doi:10.1001/jamanetworkopen.2024.3223

Data
Data available: Yes
Data types: Deidentified participant data
How to access data: Researchers meeting the criteria for access to confidential data may access the data upon request, involving the documentation of data access.
When available: With publication

Supporting Documents
Document types: None

Additional Information
Who can access the data: Researchers meeting the criteria for access to confidential data may access the data upon request, involving the documentation of data access.
Types of analyses: for any purpose
Mechanisms of data availability: with a signed data access agreement
